# Clinical and Electrophysiological Outcome Measures of Patients With Post-Infectious Neurological Syndromes Related to COVID-19 Treated With Intensive Neurorehabilitation

**DOI:** 10.3389/fneur.2021.643713

**Published:** 2021-03-12

**Authors:** Micol Avenali, Daniele Martinelli, Massimiliano Todisco, Isabella Canavero, Francesca Valentino, Giuseppe Micieli, Enrico Alfonsi, Cristina Tassorelli, Giuseppe Cosentino

**Affiliations:** ^1^Department of Brain and Behavioral Sciences, University of Pavia, Pavia, Italy; ^2^Neurorehabilitation Unit, IRCCS Mondino Foundation, Pavia, Italy; ^3^Clinical Neurophysiology Unit, IRCCS Mondino Foundation, Pavia, Italy; ^4^Emergency Neurology Unit, IRCCS Mondino Foundation, Pavia, Italy

**Keywords:** Guillain-Barrè syndrome, acute inflammatory demyelinating polyradiculoneuropathy, acute motor sensory axonal neuropathy, myelitis, neurological rehabilitation, SARS-CoV-2

## Abstract

**Background:** The clinical spectrum of coronavirus disease 2019 (COVID-19), due to severe acute respiratory syndrome coronavirus 2 (SARS-CoV-2) infection, may be quite wide, including neurological symptoms. Among them, para-infectious or post-infectious neurological syndromes (PINS), caused by an inflammatory response against the central and/or peripheral nervous system, have been reported. The aim of this paper is to illustrate the functional and neurophysiological recovery in a series of subjects with COVID-19-related PINS who underwent intensive neurorehabilitation.

**Materials and Methods:** Five patients with PINS associated with COVID-19 were evaluated at baseline and followed up for 6 months. Three of them had polyradiculoneuropathy and two patients had myelitis. The onset of the neurological syndromes was temporally associated with the SARS-CoV-2 infection. After completing the acute neurological treatments in the intensive care unit, patients underwent a personalized multidisciplinary rehabilitation program. An in-depth clinical, functional, and electrophysiological assessment was carried out at baseline and at 3- and 6-month follow-ups.

**Results:** Among patients with polyradiculoneuropathy, the electrophysiological evaluation at baseline disclosed an acute inflammatory demyelinating polyradiculoneuropathy (AIDP) in two patients and an acute motor and sensory axonal neuropathy (AMSAN) in the third patient. At follow-up, the electrophysiological features improved in one subject with AIDP and were stable in the remaining two cases. The functional assessment after neurorehabilitation showed global recovery and full independence in walking and in activities of daily life in one patient and mild improvement in the other two cases. Of the two subjects with myelitis, the baseline electrophysiological examination showed a prolonged central motor conduction time, which returned to normal in one patient, whereas it improved but remained pathological in the other patient at follow-up. The neurorehabilitation led to a substantial functional improvement in both subjects.

**Discussion and Conclusions:** This is the first study to describe clinical and electrophysiological aspects along with medium-term outcome in patients with COVID-19-related neurological manifestations who underwent an intensive rehabilitation program. The functional outcome following neurorehabilitation in patients with PINS related to SARS-CoV-2 infection is variable. In our small case series, subjects with polyradiculoneuropathy had a poorer recovery compared to patients with myelitis. The clinical course largely paralleled the follow-up electrophysiological findings.

## Introduction

Coronavirus disease 2019 (COVID-19), caused by severe acute respiratory syndrome coronavirus 2 (SARS-CoV-2) infection, is rapidly and dramatically spreading worldwide causing increasing numbers of hospitalization, intensive care admissions, and deaths (1, 2). Since the WHO declaration of pandemic on March 11, 2020, the situation is rapidly evolving, and to date, more than 6 million cases of COVID-19 have been registered globally, with severe consequences in terms of risk healthcare and collapse of economic systems (3).

Though respiratory distress and cardiovascular symptoms are the main players of the clinical picture of COVID-19, several symptoms of both central and peripheral nervous system (CNS and PNS) involvement have been related to SARS-CoV-2 infection so far (4–8).

Although a direct neurotropism and the ability of the virus to trigger an autoimmune response have been suggested (9), to date, the exact mechanisms by which SARS-CoV-2 may affect CNS and PNS still remain unclear.

The most common neurological symptoms reported in association with SARS-CoV-2 infection are dizziness, headache, ageusia, and loss of smell, while major neurological syndromes include acute cerebrovascular disease, polyradiculoneuritis [e.g., Guillain–Barré syndrome (GBS)], myelitis, acute encephalitis, meningoencephalitis, and encephalomyelitis (10–15).

Para-infectious or post-infectious neurological syndromes (PINS) associated with COVID-19 occurring shortly after the onset of respiratory symptoms are also emerging in the literature. GBS is an acute form of inflammatory polyradiculoneuropathy that often occurs after an infection or vaccination as a result of an autoimmune response triggering. GBS commonly manifests as acute ascending muscle weakness associated with sensory loss and absent or reduced deep tendon reflexes. GBS is a heterogeneous condition with several variants including forms characterized by a primary axonal injury, such as acute motor and sensory axonal neuropathy (AMSAN) and acute motor axonal neuropathy (AMAN), which can leave mid- to long-term sequelae (16, 17).

Acute myelitis is also known as a neurological complication of viral infections, which may be due to direct viral invasion or autoimmune response triggering. Little is known about the causal relationship existing between SARS-CoV-2 infection and acute myelitis, though several cases of myelitis have been reported in association with COVID-19 to date (13, 18, 19).

Neurophysiological investigations represent a fundamental tool in the early diagnosis and follow-up evaluations of both peripheral nervous system and spinal cord diseases.

Non-pharmacological strategies such as neurorehabilitative intervention could ameliorate the neurological impairment of COVID-19 patients with neurological complications. It is likely that physical therapy, when initiated in the early phases of the disease and continued in the subacute and outpatient settings, could improve the clinical outcome and quality of life of these individuals, minimizing the neurological burden and providing a better prognosis (20, 21). However, limited evidence is available on the benefit of physical therapy in the early phases of the disease, and the therapeutic effectiveness of an intensive and prolonged interdisciplinary neurorehabilitation program in patients with SARS-CoV-2-related PINS still has to be demonstrated (22, 23).

In this paper, we report a case series of five patients who developed PINS following COVID-19 infection and underwent an intensive and personalized multidisciplinary rehabilitation program. Clinical and electrophysiological findings at baseline and at 3- and 6-month follow-ups are thoroughly described.

## Materials and Methods

This is an observational study of five patients with PINS following COVID-19 who were hospitalized at the IRCCS Mondino Foundation Hospital, Pavia (Italy), in March 2020 during the first COVID-19 outbreak in Northern Italy. During the same time frame (March 2020), a total of 107 patients with other neurological conditions were admitted to the Emergency Neurology Unit and other neurological departments of our hospital. Among them, no other patients with myelitis were observed, and just another patient with GBS not associated with SARS-CoV-2 infection was hospitalized. All diagnostic investigations and treatments were carried out according to clinical needs and independently by the research aims.

In all subjects, COVID-19 infection was confirmed by means of a nasopharyngeal swab, followed by detection of serum IgG antibodies against SARS-CoV-2. No patient had previous history of central or peripheral neurological disease, cranial or spinal surgery, or traumatic head or spinal injury.

Cerebrospinal fluid (CSF) was collected and processed for standard analyses including white blood cell count, level of proteins and glucose, and real-time quantitative PCR (RT-qPCR) for SARS-CoV-2. Chest radiography and, in some patients, MRI scans were also performed at baseline.

After completing the diagnostic assessment and acute neurological treatments in the intensive care unit (ICU), subjects were admitted to the neurorehabilitation unit once they tested negative at nasopharyngeal swab SARS-CoV-2 RT-qPCR testing and were started on a personalized multidisciplinary rehabilitation program (RP). The RP consisted of six individual sessions per week, each lasting 90 min. The RP intervention involved a program of functional exercise with increasing intensity and duration of activity or exercise, dynamically adapted to progressive clinical improvement. More specifically, the program included different exercise modalities, ranging from passive mobilization, neurosensory stimulation, isokinetic muscle strengthening, active mobilization, endurance training, postural control, balance, transfers, and gait training. Subjects gave their written informed consent to all study procedures.

After completing the RP, all patients were discharged at home and returned for a clinical and laboratory follow-up at 3 and 6 months after the onset of neurological symptoms.

All patients underwent an in-depth clinical evaluation by means of the Medical Research Council's scale (MRC scale) for evaluation of muscle strength, Functional Independence Measure (FIM) scale, Barthel index, Tinetti and Hauser scale at baseline and at the follow-up visits. An extensive neurophysiological investigation was also carried out at baseline and at 3- and 6-month follow-ups, with the exception of patient #3 who performed the electrophysiological reevaluation only at 3-month after onset of the neurological symptoms.

The electrophysiological assessment included nerve conduction and electromyography (EMG) studies along with motor evoked potential (MEP) assessment. All investigations were performed by means of a five-channel electromyograph (Synergy, Medelec, UK). The following motor nerve conduction parameters of the four limbs were assessed: distal latency, amplitude of the compound muscle action potential, and conduction velocity of the common peroneal, tibial, and ulnar nerves, as well as F wave latency of the tibial and ulnar nerves. Antidromic sensory nerve conduction parameters of the limbs comprised amplitude of the sensory nerve action potential and conduction velocity of the sural and ulnar nerves. EMG of the tibialis anterior, gastrocnemius medialis, vastus lateralis, first dorsal interosseous, and deltoid muscles was performed bilaterally using concentric needle electrodes. The following EMG parameters were assessed: the presence of spontaneous activity (i.e., fibrillation potentials, positive sharp waves, and complex repetitive discharges), motor unit action potential (MUAP) analysis (i.e., duration and amplitude), and spatial recruitment of MUAPs (i.e., normal, reduced, or early interference pattern). MEPs were obtained by means of a single-pulse monophasic electromagnetic stimulator (STM9000, Ates Medica Device, EB Neuro, Italy) capable of generating a maximal output of 2.4 Tesla. Cortical and spinal hot spots for the abductor hallucis and abductor digiti minimi muscles were stimulated using a 90-mm circular coil (inner diameter of 5 cm). Magnetic pulse intensity, expressed as the percentage of the maximal stimulator output, was set up to obtain the MEP with suprathreshold amplitude size. The following MEP parameters were assessed: cortical and peripheral MEP amplitude, cortical and peripheral motor conduction time, and central motor conduction time (CMCT), defined as the difference between the cortical and peripheral motor conduction time.

The study was approved by the local ethics committee (IRCCS San Matteo Polyclinic in Pavia). Written informed consent was obtained from all subjects involved in the study.

## Results

### Baseline Evaluations

Demographic, clinical, laboratory, and instrumental findings of the patients enrolled both at baseline and at follow-up examinations are summarized in Tables 1, 2. The main rehabilitation outcomes are reported in Table 3.

**Table 1:** Demographic, and main clinical and laboratory/instrumental findings of the subjects enrolled in this case series.

**Patient**	**#1**	**#2**	**#3**	**#4**	**#5**
Age, years	61	72	57	69	25
Sex	M	F	M	M	F
Diagnosis	Acute inflammatory demyelinating polyradiculoneuropathy (AIDP)	Acute inflammatory demyelinating polyradiculoneuropathy (AIDP)	Acute motor sensory axonal neuropathy (AMSAN)	Myelitis	Myelitis
Early symptoms of COVID-19	Cough, hyposmia and dysgeusia	Fever (up to 39°C), cough, ageusia	Fever (up to 39°C), cough and dysgeusia	Fever and asthenia	Fever (up to 38°C), anosmia and dysgeusia
Need for mechanical ventilation	Yes	No	No	No	No
COVID-19 treatment	None	Antibiotics, LMWH and hydroxychloroquine	None	Antibiotics, lopinavir/ritonavir, LMWH and hydroxychloroquine	LMWH
Latency of neurological symptoms, days	21	8	12	3	15
CSF findings	Normal, SARS-CoV-2 RT-qPCR negative	1st exam: normal, SARS-CoV-2 RT-qPCR negative 2nd exam: albumin-cytological dissociation	Normal, SARS-CoV-2 RT-qPCR negative	Marked pleocytosis with neutrophil prevalence, hyperproteinorrachia and oligoclonal bands, SARS-CoV-2 RT-qPCR negative	Normal (polyclonal distribution of immunoglobulins), SARS-CoV-2 RT-qPCR negative
Brain MRI findings	Not performed	Chronic cerebrovascular disease	Not performed	Normal	Normal
Spinal cord MRI findings	–	No signs of myelitis nor thickening or contrast enhancement of nerve roots	No signs of myelitis nor thickening or contrast enhancement of nerve roots	Multiple small T2-hyperintense cervical and thoracic lesions, mostly affecting the lateral and posterior columns	T2-hyperintensity at the thoracic spinal cord level mainly affecting the T3 and T8-T10 myelomeres
Neurological symptoms at baseline	Impaired walking and sensory loss at the lower limbs which rapidly evolved to tetraparesis with acute respiratory failure	Walking impairment and diffuse paresthesia, gradually evolving in a tetraparesis with sensory deficit in the four limbs	Progressive sensory-motor deficit in the four limbs (sensory symptoms prevalent on motor impairment)	Acute urinary retention, rapidly followed by complete motor and sensory impairment in the lower limbs	Hyposthenia in both legs, paresthesia and numbness with upper level at the breast line and sensation of incomplete bladder emptying
Acute Treatments of the neurological syndrome	IVIG, 1 cycle of 3 days (0.4 g/die)	IVIG, 2 cycles of 5 days each (0.4 g/die) within the first month	IVIG, 1 cycle of 5 days (0.4 g/die)	IVIG, 1 cycle of 5 days (0.4 g/die)	2 cycles of IV methylprednisolone 1 g/day (each of 7 days) with a 3-month interval
Chronic treatments of the neurological syndrome	None	Plasma exchange cycles, 6 times over 14 days	None	None	Low dose of oral prednisone with a tapering scheme over 2 months
Duration of rehabilitation treatment including physiotherapy and occupational therapy, days	120	179	36	128	72
Clinical outcome	Autonomy recovery; persistence of mild distal weakness at lower limbs	Autonomy in self transferring, ability to walk with aids and bilateral support	Autonomy recovery; walking with right ankle-foot orthosis	Autonomy recovery; normal walking but with early fatigue	Autonomy recovery; persistence of distal weakness at lower limbs and gait ataxia

*IVIG, intravenous immunoglobulin; LMWH, low molecular weight heparin*.

**Table 2:** Detailed clinical features of patients at baseline and at 3- and 6-month follow-ups.

**Patient**	**Baseline**				**3-month follow-up**				**6-month follow-up**			
	**Deep tendon reflexes**	**Sensory symptoms and signs**	**MRC scale Upper limbs: R/L**	**MRC scale Lower limbs: R/L**	**Deep tendon reflexes**	**Sensory symptoms and signs**	**MRC scale Upper limbs: R/L**	**MRC scale Lower limbs: R/L**	**Deep tendon reflexes**	**Sensory symptoms and signs**	**MRC scale Upper limbs: R/L**	**MRC scale Lower limbs: R/L**
#1	Absent in the lower and upper limbs, normal plantar reflex response bilaterally	Severe hypoesthesia in the lower limbs till the superior anterior iliac spine	39/35	19/21	Hyporeflexia only in the lower limbs, normal plantar reflex response bilaterally	Moderate hypoesthesia at the lower limbs	24/24	9/9	Areflexia of the Achilles reflexes; DPR normalized in the other districts; normal plantar reflex response bilaterally	Mild hypoesthesia in the lower limbs, till the ankle	42/41	18/18
#2	Hyporeflexia in the upper limbs, areflexia in the lower limbs, silent plantar reflex response bilaterally	Distal hypoesthesia and paresthesia in the four limbs	21/23	6/8	Hyporeflexia in the upper limbs, areflexia in the lower limbs, silent plantar reflex response bilaterally	Slight improvement of the distal hypoesthesia and paresthesia in the four limbs	22/24	6/9	Hyporeflexia in the lower limbs, abnormal plantar reflex response bilaterally	Mild distal paraesthesia in the lower limbs	28/29	13/14
#3	Hyporeflexia of the patellar reflexes, areflexia of the Achilles tendon reflexes, silent plantar reflex response bilaterally	Distal hypoesthesia in the four limbs more prominent in the right lower limb below the knee; distal apallesthesia in the lower limbs	41/41	12/25	Areflexia of the Achilles reflexes, silent plantar reflex response bilaterally	Mild recovery of the hypoesthesia now confined to the right lower limb above the knee and at the left foot; no variation in the distal apallesthesia in the lower limbs	42/38	17/19	NA	NA	NA	NA
#4	Hyporeflexia in the upper limbs, areflexia in the lower limbs, bilateral Babinski sign	Severe hypoesthesia and hypopallesthesia in the lower limbs	40/40	9/6	Hyperreflexia in the lower limbs, bilateral Babinski sign	Mild hypoesthesia in the lower limbs	40/40	21/20	Mild hyperreflexia at lower limbs, bilateral Babinski sign	Normal	40/40	24/24
#5	Hyperreflexia of the patellar and Achilles tendon reflexes, bilateral Babinski sign	Severe hypoesthesia and hypopallesthesia in the lower part of the body with upper level at T4-T5	40/40	16/16	Hyperreflexia of the patellar and Achilles reflexes with ankle clonus; bilateral Babinski sign	Moderate improvement of the hypoesthesia, upper level at T4-T5	40/40	19/20	Hyperreflexia of the patellar and Achilles reflexes with ankle clonus; bilateral Babinski sign	Mild hypoesthesia with upper level T4-T5	40/40	24/24

*DPR, Deep Tendon Reflexes; MRC, Medical Research Council Scale for muscle strength assessment. Muscle effort evaluated in the upper limbs (sum score ranging between 0 and 40 for each body side) included: deltoid, biceps brachii, triceps brachii, wrist extension, wrist flexion, finger extension, finger flexion, first dorsal interosseous muscles. Muscle effort evaluated in the lower limbs (sum score ranging between 0 and 25 for each body side) included: iliopsoas, quadriceps femoralis, gastrocnemius, tibialis anterior, extensor hallucis longus mucles*.

**Table 3:** Functional status of the subjects at baseline and follow-ups.

**Patient**	**Barthel index**			**FIM**			**Hauser**			**Tinetti**		
	**Baseline**	**3-m FU**	**6-m FU**	**Baseline**	**3-m FU**	**6-m FU**	**Baseline**	**3-m FU**	**6-m FU**	**Baseline**	**3-m FU**	**6-m FU**
#1	0	50	95	10	56	115	9	7	2	0	12	26
#2	30	30	35	44	47	76	9	9	7	0	1	11
#3	60	80	NA	85	115	NA	9	3	NA	6	23	NA
#4	35	70	90	69	105	121	9	3	2	0	18	28
#5	65	90	100	95	108	110	5	5	2	15	17	26

*Barthel, Barthel index for activities of daily living (0–100); FIM, Functional Independence Measure index (0–126); Hauser, Hauser ambulatory index (0–9); Tinetti, Tinetti assessment tool, balance plus gait score (0–28); 3-m FU, follow-up at 3 months; 6-m FU, follow-up at 6 months*.

All subjects were admitted to the neurorehabilitation unit with mild motor signs in the lower limbs and variable sensory involvement (Tables 1, 2, 4). The baseline electrophysiological study was consistent with a diagnosis of GBS in patients #1, #2, and #3. In particular, according to Uncini's criteria (24), nerve conduction findings were compatible with an acute inflammatory demyelinating polyradiculoneuropathy (AIDP) variant in patients #1 and #2 and with an AMSAN in patient #3. Nerve conduction investigation disclosed signs of a severe and widespread axonal damage in all three subjects. Accordingly, EMG showed very rich spontaneous activity (fibrillation potentials and positive sharp waves) and severely reduced spatial recruitment of MUAPs bilaterally in the tibialis anterior, gastrocnemius medialis, and first dorsal interosseous muscles of all three patients. The spatial recruitment of MUAPs was reduced, albeit to a lesser extent, in the vastus lateralis and deltoid muscles on both sides, while MUAP parameters were within normal limits in all muscles.

**Table 4:** Nerve conduction findings in subjects with polyradiculoneuropathy.

**Nerves**	**ENG parameters**	**Patient #1**			**Patient #2**			**Patient #3**		**Normal values**
		**Baseline**	**3-month follow-up**	**6-month follow-up**	**Baseline**	**3-month follow-up**	**6-month follow-up**	**Baseline**	**3-month follow-up**	
**Common peroneal**										
Ankle – EDB	DL, ms	R = 12.7; L = 11.8	–	–	–	–	–	R = –; L = 4.0	R = –; L = 4.6	≤6.5
	Amp, mV	R = 0.6; L = 0.7	R = Abs; L = Abs	R = Abs; L = Abs	R = Abs; L = Abs	R = Abs; L = Abs	R = Abs; L = Abs	R = Abs; L = 0.1	R = Abs; L = 0.1	≥2
Below fibula – Ankle	Amp, mV	R = 0.1; L = 0.1	R = Abs; L = Abs	R = Abs; L = Abs	R = Abs; L = Abs	R = Abs; L = Abs	R = Abs; L = Abs	R = Abs; L = 0.1	R = Abs; L = 0.1	≥2
	CV, m/s	R = 19.2; L = 20.3	–	–	–	–	–	R = –; L = 36.7	R = –; L = 34.9	≥44
**Tibial**										
Ankle – AH	DL, ms	R = 20.7; L = 17.6	R = 13.1; L = 9.4	R = 6.7; L = 6.2	R = 6.4; L = 4.8	R = 7.2; L = 4.7	R = 7.5; L = 4.6	–	–	≤5.8
	Amp, mV	R = 0.3; L = 0.6	R = 0.5; L = 0.7	R = 0.6; L = 0.8	R = 0.4; L = 0.8	R = 0.3; L = 0.4	R = 0.1; L = 0.1	R = Abs; L = Abs	R = Abs; L = Abs	≥4
	F latency, ms	R = Abs; L = Abs	R = 95.8; L = 84.6	R = 95.2; L = 84.0	R = Abs; L = Abs	R = Abs; L = Abs	R = Abs; L = Abs	R = Abs; L = Abs	R = Abs; L = Abs	≤52
Popliteal fossa – Ankle	Amp, mV	R = 0.1; L = 0.1	R = 0.4; L = 0.6	R = 0.4; L = 0.8	R = 0.4; L = 0.8	R = 0.3; L = 0.4	R = 0.1; L = 0.1	R = Abs; L = Abs	R = Abs; L = Abs	≥4
	CV, m/s	R = 16.9; L = 17.5	R = 27.5; L = 28.3	R = 29.6; L = 30.4	R = 39.1; L = 39.3	R = 37.8; L = 37.2	R = 35.0; L = 36.7	–	–	≥41
**Ulnar (motor)**										
Wrist – ADM	DL, ms	R = 8.8; L = 8.7	R = 5.3; L = 5.1	R = 3.5; L = 4.0	R = 4.2; L = 3.6	R = 4.4; L = 3.8	R = 4.5; L = 3.9	R = 2.2; L = 2.5	R = 3.2; L = 2.7	≤3.3
	Amp, mV	R = 0.8; L = 0.6	R = 8.6; L = 6.3	R = 9.7; L = 7.4	R = 1.5; L = 1.6	R = 1.6; L = 1.7	R = 1.8; L = 2.9	R = 8.5; L = 8.9	R = 7.0; L = 8.1	≥6
	F latency, ms	R = 57.2; L = 59.4	R = 40.3; L = 39.4	R = 39.0; L = 37.0	R = Abs; L = Abs	R = 41.8; L = 38.4	R = 40.2; L = 36.7	R = 30.8; L = 30.5	R = 31.9; L = 34.2	≤30
Below elbow – Wrist	Amp, mV	R = 0.8; L = 0.6	R = 8.5; L = 6.2	R = 7.1; L = 7.3	R = 0.7; L = 0.8	R = 1.5; L = 1.6	R = 1.5; L = 2.4	R = 7.0; L = 6.7	R = 5.1; L = 5.5	≥6
	CV, m/s	R = 25.1; L = 24.2	R = 37.5; L = 39.4	R = 42.6; L = 41.9	R = 34.0; L = 35.8	R = 35.1; L = 36.7	R = 35.4; L = 38.8	R = 54.4; L = 59.2	R = 56.0; L = 50.0	≥49
**Sural**										
Calf – Posterior ankle	Amp, μV	R = 1.5; L = 0.6	R = 1.7; L = 1.3	R = 1.9; L = 1.4	R = 4.4; L = 0.6	R = 1.4; L = 0.4	R = 0.3; L = 0.2	R = Abs; L = Abs	R = Abs; L = Abs	≥6
	CV, m/s	R = 27.4; L = 24.2	R = 30.5; L = 30.2	R = 31.2; L = 31.6	R = 35.7; L = 32.8	R = 36.4; L = 36.0	R = 29.0; L = 35.1	–	–	≥40
**Ulnar (sensory)**										
Wrist – Digit 5	Amp, μV	R = 4.6; L = 5.8	R = 7.4; L = 7.9	R = 9.8; L = 11.3	R = 1.0; L = 4.2	R = 3.9; L = 4.8	R = 6.7; L = 9.3	R = 6.9; L = 1.6	R = 2.7; L = 0.9	≥10
	CV, m/s	R = 32.9; L = 33.2	R = 33.3; L = 34.4	R = 34.2; L = 34.6	R = 31.9; L = 22.9	R = 32.7; L = 28.0	R = 33.8; L = 34.8	R = 52.2; L = 46.2	R = 41.0; L = 41.9	≥50

*ADM, abductor digiti minimi muscle; AH, abductor hallucis muscle; Amp, amplitude; CV, conduction velocity; DL, distal latency; EDB, extensor digitorum brevis muscle; ENG, electroneurography; L, left; R, right. Normative values were defined by examining a group of 30 healthy subjects*.

Two of the three patients underwent a lumbosacral MRI scan with contrast that showed no signs of myelitis nor thickening or contrast enhancement of the nerve roots. No patient presented a positive RT-qPCR for SARS-CoV-2 on CSF samples. At symptom's onset, chest radiographies were negative for pneumonia in all patients. They were all treated with intravenous immunoglobulin (IVIG) (0.4 g/day for at least 5 days), but patients #1 and #3 presented severe worsening of the motor and sensory deficit within the first days of treatment. Patient #1 required ventilatory support and a tracheostomy due to acute respiratory failure in the context of a severe tetraparesis with axial muscle involvement and *Acinetobacter baumannii* concomitant infection. This patient required a total stay of 56 days in the ICU and started an intensive rehabilitation program only 2 months after the onset of symptoms.

Patient #3 presented severe worsening of the respiratory muscular performance as well: at first, the possibility of a plasma exchange was evaluated, but in order to avoid a possible worsening of the COVID-19 infection, we chose to perform a second IVIG cycle. The procedure halted clinical deterioration, and the patient was therefore transferred to the neurorehabilitation unit.

Patients #4 and #5 presented with progressive sensory and motor deficits in the lower limbs associated with bladder dysfunction with urinary retention (Tables 1, 2). As mentioned above, no sign of SARS-CoV-2 replication was observed in the CSF, while both patients presented altered MRI signals proving an inflammatory spinal cord involvement. Findings from electroneurography (ENG) and EMG assessment of the four limbs were within normal ranges. Both subjects tested negative for antibodies against AQP4, MOG, GQ1b, or GD1b. MEP investigation in patient #4 revealed an impaired corticospinal conduction deriving from the lower limbs, with asymmetric involvement (predominant on the left side); MEP findings in patient #5 were consistent with a diffuse impairment of the corticospinal tract, predominant when deriving from the right limbs (Table 5). After a diagnosis of myelitis was made, adequate treatment with a high dose of IV methylprednisolone (7 g in total) was performed.

**Table 5:** Motor evoked potentials findings in subjects with myelitis.

**Muscles**	**MEP parameters**	**Patient #4**			**Patient #5**			**Normal values**
		**Baseline**	**3 month follow-up**	**6 month follow-up**	**Baseline**	**3-month follow-up**	**6-month follow-up**	
AH	Cortical Amp, mV	R = 0.4; L = Abs	R = 2.1; L = 1.9	R = 3.4; L = 3.3	R = 0.1; L = 0.2	R = 0.7; L = 0.8	R = 1.0; L = 1.0	≥3.0
	Peripheral Amp, mV	R = 6.4; L = 6.1	R = 5.7; L = 5.9	R = 5.4; L = 5.7	R = 5.2; L = 6.4	R = 6.0; L = 6.8	R = 6.2; L = 7.5	≥4.0
	CMCT, ms	R = 16.9; L = –	R = 15.3; L = 17.3	R = 14.6; L = 16.4	R = 31.7; L = 24.3	R = 27.8; L = 23.1	R = 24.9; L = 17.3	≤16.7
	PMCT, ms	R = 24.3; L = 23.4	R = 23.5; L = 22.9	R = 22.7; L = 22.5	R = 23.9; L = 23.0	R = 24.1; L = 22.9	R = 24.2; L = 22.6	≤29.5
ADM	Cortical Amp, mV	R = 4.4; L = 4.2	R = 4.3; L = 4.3	R = 4.3; L = 4.2	R = 3.1; L = 3.7	R = 4.4; L = 4.7	R = 4.8; L = 5.4	≥4.0
	Peripheral Amp, mV	R = 6.2; L = 6.3	R = 5.9; L = 5.9	R = 5.9; L = 5.8	R = 7.1; L = 8.3	R = 7.3; L = 8.2	R = 7.4; L = 8.8	≥5.0
	CMCT, ms	R = 6.0; L = 5.7	R = 5.3; L = 4.9	R = 4.8; L = 4.5	R = 12.8; L = 11.0	R = 10.6; L = 8.6	R = 8.6; L = 7.4	≤6.7
	PMCT, ms	R = 14.7; L = 14.6	R = 14.4; L = 14.0	R = 14.1; L = 13.6	R = 14.0; L = 14.2	R = 13.6; L = 14.4	R = 13.4; L = 14.6	≤16.5

*ADM, abductor digiti minimi muscle; AH, abductor hallucis muscle; Amp, amplitude; CMCT, central motor conduction time; MEP, motor evoked potentials; PMCT, peripheral motor conduction time. Normative values were defined by examining a group of 30 healthy subjects*.

### Follow-Up Evaluations

At the electrophysiological follow-up, patient #1 presented with improvement of the nerve conduction parameters, especially in the upper limbs with exception for common peroneal nerves. In parallel, EMG showed disappearance of spontaneous activity, enhancement of MUAP recruitment, and neurogenic MUAPs (increased amplitude and duration) as an expression of reinnervation phenomena. From a clinical point of view, the patient globally improved except for mild hypoesthesia persisting in the lower limbs. Independence in walking and in daily living activities was fully recovered within 6 months.

Conversely, nerve conduction findings did not significantly improve in patient #2, with exception of the upper limbs' parameters, and in patient #3. In these subjects, EMG findings confirmed the remarkable axonal impairment, with persistent spontaneous activity in the same muscle districts previously examined (which was enriched by frequent complex repetitive discharges) that also involved the vastus lateralis muscle bilaterally. In both patients #2 and #3, MUAP recruitment did not significantly improve and MUAPs presented with neurogenic features in proximal and distal muscle districts of the four limbs. Nerve conduction findings at baseline and follow-up of all patients are listed in Table 4.

Taking into account these findings, since patient #2 presented with no significant lower limb motor improvement at 3-month follow-up, her case was further reevaluated. Also considering the presence of persisting active denervation in both lower limbs, she was therefore treated with six cycles of therapeutic plasma exchange over a 14-day period, with clear benefit. At 6-month follow-up, the patient has recovered short-distance walking ability (12 m) with the support of walking aids.

The clinical course of patient #3 was complicated by the occurrence of a deep venous thrombosis affecting the left leg about 2 months after the onset of neurological symptoms. This determined a reduced mobility of the left lower limb as shown by reduced MRC subscore of the left lower limb at 3-month follow-up (Table 2). The patient did not return to the 6-month follow-up, and no further information were available.

Patients #4 and #5 presented with global functional and electrophysiological improvement (Tables 2, 3, 5). From a clinical point of view, both patients reacquired full independence in daily living activities within 6 months, with persistence of minor gait abnormalities that did not require any aids. However, it is noteworthy that patient #5 presented with transient worsening of the motor deficit, leading to increased disability during the first trimester, as soon as the corticosteroid therapy was tapered. In agreement with clinical findings, signs of active progression of the disease were observed at a whole-spine MRI (increased number of lesions in both cervical and dorsal spinal cord, with tendency to confluence, without areas of pathological spinal enhancement). A second cycle of high-dose IV methylprednisolone was performed, which rapidly led to global clinical improvement. MEP findings of both patients with myelitis improved at the follow-up assessment, although in patient #5, abnormalities were still found (Table 5).

## Discussion

In this paper, we describe clinical and electrophysiological features of five patients with PINS associated with COVID-19 who underwent an intensive personalized rehabilitation program and were followed up for a 6-month period.

GBS cases associated with SARS-CoV-2 infection have been described so far (13, 14). GBS is an autoimmune syndrome characterized by inflammatory axonal and/or demyelinating neuropathy. It may lead to severe sequelae, disability, or even death when a severe neuromuscular respiratory failure occurs. Depending on the GBS subtype (e.g., axonal vs. demyelinating damage), the outcomes are largely variable, ranging from poor recovery to remarkable improvement (11). When GBS is associated with SARS-CoV-2 infection, the recovery may be complicated by an overlap with severe respiratory symptoms, leading to a worse prognosis. Indeed, patients #1 and #3 presented in the early stages severe worsening of the respiratory performance, requiring urgent ventilatory support; in patient #1, tracheostomy was indeed required and the pulmonary function was further impaired by a severe bacterial lung infection. In this regard, it should be noted that none of the patients showed signs of COVID-19-related pneumonia at chest X-ray, though it is likely that some degree of pulmonary damage might have been detected by a CT scan of the chest, but such an investigation was not performed.

Similarly to other acute neurological conditions, even patients with GBS and myelitis related to COVID-19 may benefit from a neurorehabilitation intervention begun in the early phases after onset of neurological symptoms in terms of fostering recovery and determining a better prognosis.

In this study, all subjects underwent an intensive rehabilitation program, personalized according to their level of disability and progressively incremented after improvement in their performances was observed.

At baseline, all patients with polyradiculoneuropathy presented signs of considerable axonal damage at the electrophysiological assessment that could probably explain the poorer prognosis with respect to the patients with myelitis. The two subjects with AIDP variant of GBS had a different clinical and electrophysiological outcome. In patient #1, both clinical measures and electrophysiological parameters improved at the 6-month follow-up, and the patient reacquired functional autonomy and was able to walk independently with only slight distal weakness persisting in the lower limbs. In patient #2, despite the prolonged rehabilitation intervention (179 days), relevant neurological deficits remained, although the patient reacquired the ability to walk short distances with aids and bilateral support. The electrophysiological assessment confirmed the presence of a remarkable axonal impairment even at the follow-up. Patient #3, affected by an AMSAN GBS variant, presented with a rapid clinical improvement in the first months in the absence of significant amelioration of ENG and EMG parameters at 3-month follow-up. This apparent discrepancy was likely linked also to the relatively short time period from the previous instrumental evaluation. Unfortunately, the clinical status of the patient was further deteriorated by a deep venous thrombosis in the left leg, which led to reduced mobility.

Within our limited case series, we observed that two out of three GBS patients developed axonal forms of the disease, contrary to what was reported by Filosto et al. (25), who instead reported a higher prevalence of COVID-related demyelinating forms of GBS in a broader case series. However, data from the literature are still too preliminary to understand whether the clinical and prognostic profile of GBS or myelitis related to SARS-CoV-2 infection may present peculiar features.

The two subjects with myelitis showed global clinical improvement after the RP that was in line with the results of MEP assessment at the follow-up.

Major limitations of this study are represented by the small number of cases enrolled and by the clinical heterogeneity of the SARS-CoV-2 infection-related PINS, which involved either the PNS or the CNS. However, no other myelitis and just one patient with GBS not COVID-19-related among 107 patients was hospitalized at Mondino Foundation during the pandemic peak that hit Northern Italy in March 2020. We are also aware that the absence of a control group may have limited this study. However, in this context, the control condition (being no rehabilitation) is considered an unethical option for patients with functional limitations caused by PINS.

Notwithstanding, a novelty of this study is represented by the in-depth description of clinical and electrophysiological aspects of patients with rare neurological manifestations of COVID-19 who underwent an intensive rehabilitation program and were followed up for a relatively long time period of 6 months. Current literature on COVID-19 is mainly focused on clinical manifestations and complications of the SARS-CoV-2 infection in the acute phase, while evidence regarding long-term outcome is still lacking (22, 26, 27). To our knowledge, this is the first report to suggest the important role of neurological rehabilitation intervention in COVID-19 patients with neurological impairment.

## Data Availability Statement

The raw data supporting the conclusions of this article will be made available by the authors, without undue reservation.

## Ethics Statement

The studies involving human participants were reviewed and approved by Ethics Committee of the IRCCS San Matteo Polyclinic in Pavia. The patients/participants provided their written informed consent to participate in this study.

## Author Contributions

MA, DM, and MT: drafting of manuscript. EA, CT, and GC: study conception and design. MA, DM, MT, IC, FV, and GM: acquisition of data. MA, DM, MT, and GC: analysis and interpretation of data. All authors critical revision.

## Conflict of Interest

The authors declare that the research was conducted in the absence of any commercial or financial relationships that could be construed as a potential conflict of interest. The handling editor declared a past co-authorship, with one of the authors with several of the authors CT, GC, DM, MT, EA, and MA.
